# Effects of perfusion pressure on the splanchnic circulation after cardiopulmonary bypass: a randomized double cross-over study

**DOI:** 10.1186/cc13376

**Published:** 2014-03-17

**Authors:** L McNicol, M Lipcsey, R Bellomo, F Parker, S Poustie, G Liu, S Uchino, A Kattula

**Affiliations:** 1 Austin Hospital, Heidelberg, Australia; 2 Uppsala University, Uppsala, Sweden; 3 Jikei University, Tokyo, Japan

## Introduction

No randomized trial has assessed the effects of different mean arterial pressure (MAP) targets in postcardiac surgery intensive care. We investigated the short-term effects of MAP of 65 or 85 mmHg on splanchnic oxygen flux, metabolic function, mucosal perfusion and cytokine regulation.

## Methods

A single-center, randomized controlled, double cross-over trial was performed. Patients were randomized to: HLH (high-low- high) where MAP targets were 85-65-85 mmHg in sequence, with each lasting 2 hours, or LHL (low-high-low) where MAP targets were 6585-65 mmHg. Blood pressure was adjusted with noradrenalin infusion.

## Results

Six + six patients were included in the study. MAP targets were achieved in all patients at all time points (64 ± 3, 84 ± 4; 65 ± 5 mmHg in the LHL group and 84 ± 3; 66 ± 2; 85 ± 5 mmHg in the HLH group at the first, second and third time points), with corresponding changes in filling pressures. Cardiac output did not change over time. Hepatic venous saturation was 41 ± 15; 58 ± 24; 56 ± 21% in the LHL group and 50 ± 19; 43 ± 20; 41 ± 18% in the HLH group at the first, second and third time points, with a significant time group interaction (*P *< 0.05). No changes were observed in global or trans-splanchnic lactate levels and cytokine levels or in gastric tonometry CO_2_.

## Conclusion

Increasing MAP with norepinephrine has some effects splanchnic oxygenation, but has no impact on metabolic or biochemical function and key cytokine removal or release. MAP targets of 60 to 65 mmHg or 80 to 85 mmHg appear physiologically equivalent for the splanchnic circulation.

